# The intra‐arterial selective cooling infusion system: A mathematical temperature analysis and in vitro experiments for acute ischemic stroke therapy

**DOI:** 10.1111/cns.13883

**Published:** 2022-06-15

**Authors:** Miaowen Jiang, Ming Li, Yuan Gao, Longfei Wu, Wenbo Zhao, Chuanhui Li, Chengbei Hou, Zhengfei Qi, Kun Wang, Shiqiang Zheng, Zhichen Yin, Chuanjie Wu, Xunming Ji

**Affiliations:** ^1^ School of Instrumentation and Optoelectronic Engineering Beihang University Beijing China; ^2^ Beijing Institute of Geriatrics, Xuanwu Hospital Capital Medical University Beijing China; ^3^ Department of Neurosurgery, Xuanwu Hospital Capital Medical University Beijing China; ^4^ Center for Evidence‐Based Medicine, Xuanwu Hospital Capital Medical University Beijing China; ^5^ Beijing Institute for Brain Disorders Capital Medical University Beijing China; ^6^ BUAA‐CCMU Advanced Innovation Center for Big Data‐based Precision Medicine Beihang University Beijing China

**Keywords:** autologous blood, ischemic stroke, numerical analysis, prototype instrument, therapeutic hypothermia

## Abstract

**Introduction:**

The neuroprotection of acute ischemic stroke patients can be achieved by intra‐arterial selective cooling infusion using cold saline, which can decrease brain temperature without influencing the body core temperature. This approach can lead to high burdens on the heart and decreased hematocrit in the scenario of loading a high amount of liquid for longtime usage. Therefore, autologous blood is utilized as perfusate to circumvent those side effects.

**Methods:**

In this study, a prototype instrument with an autologous blood cooling system was developed and further evaluated by a mathematical model for brain temperature estimation.

**Results:**

Hypothermia could be achieved due to the adequate cooling capacity of the prototype system, which could provide the lowest cooling temperature into the blood vessel of 10.5°C at 25 rpm (209.7 ± 0.8 ml/min). And, the core body temperature did not alter significantly (−0.7 ~ −0.2°C) after 1‐h perfusion. The cooling rate and temperature distributions of the brain were analyzed, which showed a 2°C decrease within the initial 5 min infusion by 44 ml/min and 13.7°C perfusate.

**Conclusion:**

This prototype instrument system could safely cool simulated blood in vitro and reperfuse it to the target cerebral blood vessel. This technique could promote the clinical application of an autologous blood perfusion system for stroke therapy.

## Graphical abstract

The safety and efficacy of the intra‐arterial selective cooling infusion system were preliminarily evaluated by mathematical brain temperature estimation and in vitro experiment. This technique can circumvent the adverse effects of saline perfusion and promote the clinical application of an autologous blood perfusion system for stroke therapy. This system is a reliable tool for neuroprotective studies on stroke animal models.

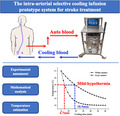

## 1 INTRODUCTION

Stroke is one of the leading causes of mortality and disability worldwide.^1^ Acute ischemic stroke (AIS) accounts for approximately 80% of all strokes.^2^ The clinical prognosis of patients with AIS can be improved by vascular recanalization via intravenous thrombolysis or mechanical thrombectomy to restore cerebral blood perfusion of the ischemic brain region.^3^ However, vascular recanalization does not guarantee a positive outcome. More than 80% of patients with acute occlusion of large cerebral arteries cannot be recanalized by using those endovascular techniques, but over 70% of patients die or remain disabled for the rest of their lives.^4^, ^5^ This can be solved by using neuroprotective strategies.

Therapeutic hypothermia (TH) is one of the well‐researched, effective, and promising neuroprotective strategies for AIS therapy. It is also the only effective neuroprotective treatment in patients with hypoxic brain injury following cardiac arrest.^6^, ^7^ Systemic hypothermia such as surface cooling lowers both the brain temperature and core body temperature. The drop in core body temperature causes a cascade of negative side effects that counteract the hypothermia treatment's efficacy^8^，such as pneumonia (40%), electrolyte problems (50%), and arrhythmias and heart failure (80%), which has limited the application of systemic hypothermia for AIS treatment.^9^ As a result, local hypothermia by selective brain cooling (SBC) has evolved into a viable and promising neuroprotective treatment.^10^

In the previous study, our group employed targeted local hypothermia in brain tissues of MCAO rodent animal models by intra‐arterial selective cooling saline infusion (IA‐SCI), which decreased cerebral infarction volume and enhanced neurological performance.^11^, ^12^ Then, in a non‐human primate model of embolic stroke, we found that IA‐SCI improved its long‐term neurological outcomes.^13^ And in clinical investigations, we verified that IA‐SCI was safe^14^ and could further reduce the expansion of cerebral infarction AIS patients on the basis of intravascular thrombolysis or mechanical thrombectomy.^15^ Except for its beneficial effects, the current dilemma of IA‐SCI is that it will increase body fluid volume, can increase heart strain and lower hematocrit, and cannot be exploited for long‐term usage.^8^

Using cold autologous blood instead of normal saline under cardiopulmonary bypass is an appropriate way of solving the abovementioned negative effects of IA‐SCI.^16^ In a rat model of cerebral infarction, we have preliminarily shown that the intra‐arterial selective cooling autogenous blood infusion (IA‐SCAI) is both safe and efficacious.^17^

Currently, there are currently no autologous blood cooling infusion systems designed specifically in clinical for cerebral hypothermia therapy, and the optimized hypothermic perfusion parameters are unavailable. In order to achieve clinical transformation and long‐term targeted local hypothermia maintenance of IA‐SCAI, a prototype instrument system for IA‐SCAI was designed and constructed in this study, which consisted of hemodynamic driver, heat exchanger, extracorporeal circulation lines, and monitoring devices. For the purpose of evaluating the cooling effect during the process of IA‐SCAI by using this system, a brain temperature estimation of hemodynamics and biological heat transfer based on numerical simulations and in vitro experiments were also proposed. The temperature of cold autogenous blood in the IA‐SCAI system at varied flow rates was acquired through an arterial analog vascular system, which was built by three‐dimensional (3D) modeling and 3D‐printing technology.

Based on the Pennes' biological heat transfer mathematical model,^18^ some optimized mathematical models were used to evaluate and predict brain temperature and its influencing factors under the condition of different hypothermia therapy methods.^19^, ^20^, ^21^, ^22^, ^23^, ^24^ In this study, perfusion flow rates, metabolism‐produced heat, and brain tissue depth were taken into consideration during the simulation. We obtained temperature response curves with different cooling power and cooling time and confirmed the feasibility of the approach in the treatment of hypothermia for AIS therapy by using the IA‐SCAI prototype instrument system.

## 2 MATERIALS AND METHODS

### 2.1 The IA‐SCAI prototype instrument system

**FIGURE 1:**
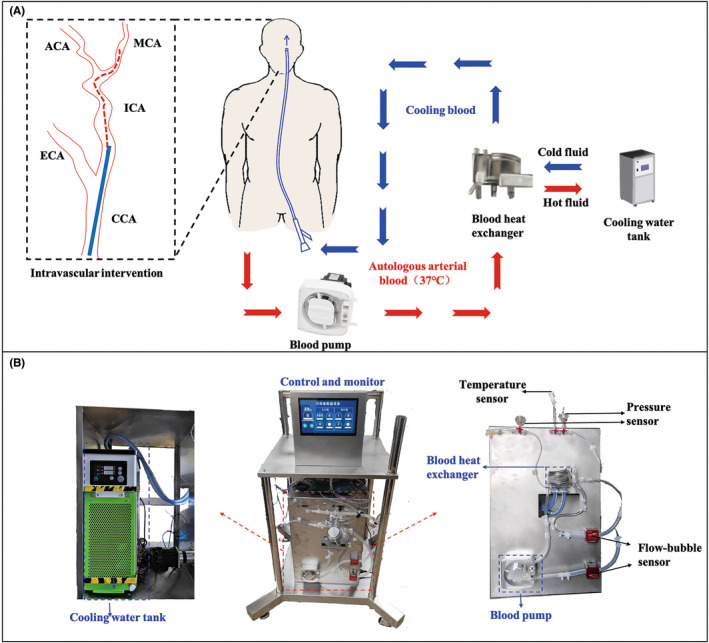
(A) Schematic diagram of the IA‐SCAI process (Common carotid artery: CCA; External carotid artery: ECA; Internal carotid artery: ICA; Middle cerebral artery: MCA; Anterior cerebral artery: ACA). (B) The prototype instrument system for IA‐SCAI

Based on extracorporeal circulation technology, in this IA‐SCAI hypothermia system, the patient's autologous blood is exported from the femoral artery using a blood pump, then it is cooled by a heat exchanger, and reperfused into the target brain tissue via the internal carotid artery (ICA). (Figure 1A) A customized circulation device is used to duplicate the IA‐SCAI process, which includes a blood peristaltic pump (MP300; Prefluid), blood heat exchanger (MYOtherm XP; Medtronic), cooling water tank (T1; Auwii), extracorporeal circuit, an intervention cooling catheter (Codman ENVOY® Catheter, 5F), temperature sensors (HEL‐705‐U‐1‐12‐00; Honeywell), blood pressure sensors (ABPDANN160KGAA5; Honeywell), and flow‐bubble sensors (CO.56; Sonotec). (Figure 1B).

### 2.2 Theoretical model for brain temperature prediction

In the event of restricted nondestructive intracranial temperature monitoring methods, theoretical computational modeling of brain tissue thermal analysis has become a significant tool for predicting brain tissue temperature in IA‐SCAI. The Pennes' bioheat biological heat transfer equation may be used to calculate *T* for brain tissue temperature.^20^ Heat conduction in tissue, heat generation through metabolism, and heat exchange between arterial blood and tissue are all taken into account in this equation.

(1) $$\rho_{t}c_{t}\frac{\partial T}{\partial t}=\nabla \left(\lambda_{t}\nabla T\right)+\rho_{b}\cdot c_{b}\cdot \omega \cdot \left(T_{a}-T\right)+P_{\mathrm{Met}}$$

where $\rho_{t}\left(\mathrm{kg}/m^{3}\right)$ is the tissue density; $c_{t}\left(J/\mathrm{Kg}\cdot K\right)$ is the specific heat; $\lambda_{t}\left(W/K\cdot m\right)$ is the thermal conductivity; $c_{b}\left(J/\mathrm{Kg}\cdot K\right)$ is the specific heat of blood; $\rho_{b}\left(\mathrm{kg}/m^{3}\right)$ is the blood density; $\omega \;\left(\mathrm{ml}/\min\cdot 100\;g\right)$ is the perfusion rate; $T_{a}\left({}^{{}^{\circ}}C\right)$ is the blood temperature of the perfusion; $P_{\mathrm{Met}}\left(W/m^{3}\right)$ is the metabolic heat of the tissue. The effect of cooling time (*t*) and brain tissue depth (*r*) on temperature change during IA‐SCAI was investigated based on the Pennes’ equation (Equation 1). The metabolic thermogenesis of brain tissue (gray matter and white matter) was taken into account in the development of a theoretical computer model (Equation 2) for brain temperature prediction.

(2) $$\rho_{t}c_{t}\frac{\partial T}{\partial t}=\frac{\lambda_{t}}{r^{2}}\cdot \frac{\partial }{\partial r}\left(r^{2}\cdot \frac{\partial T}{\partial r}\right)+\rho_{b}\cdot c_{b}\cdot \omega \cdot \left(T_{a}-T\right)+P_{\mathrm{Met}}$$

Michenfelder and Milde et al. hypothesized that the metabolic heat generation rate dropped by a factor of 3.^25^ Following that, Xu et al. proposed an analytical formula (Equation 3) for metabolic heat production based on brain temperature as a criterion^26^:

(3) $$P_{\mathrm{Met}}=q_{0}\times 3^{\left(0.1T-3.7\right)}$$

*q*_0_ is the metabolic rate of brain tissue. To solve the mathematical model (Equation 2), we employ the finite element approach and the numerical difference method:

(4) $$\frac{\partial T}{\partial t}=\frac{T\left(i+1,j\right)-T\left(i,j\right)}{\rho_{t}\cdot c_{t}\cdot \mathrm{\Delta t}}=\frac{1}{\rho_{t}\cdot c_{t}\cdot \mathrm{\Delta t}}\left\{\frac{\lambda_{t}}{r^{2}}\cdot \frac{\partial }{\partial r}\cdot \left[r^{2}\cdot \frac{\partial T\left(i+1,j\right)-\partial T\left(i,j\right)}{\partial r}\right]+\rho_{b}\cdot c_{b}\cdot \omega \cdot \left[T_{a}-T\left(i+1,j\right)+T\left(i,j\right)\right]+q_{0}\times 3^{\left(0.1T-3.7\right)}\right\}$$

(5) $$\frac{\partial T}{\partial r}=\frac{T\left(i,j+1\right)-T\left(i,j\right)}{\rho_{t}\cdot c_{t}\cdot \mathrm{\Delta r}}=\frac{1}{\rho_{t}\cdot c_{t}\cdot \mathrm{\Delta r}}\left\{\frac{\lambda_{t}}{r^{2}}\cdot \frac{\partial }{\partial r}\cdot \left[r^{2}\cdot \frac{\partial T\left(i,j+1\right)-\partial T\left(i,j\right)}{\partial r}\right]+\rho_{b}\cdot c_{b}\cdot \omega \cdot \left[T_{a}-T\left(i,j+1\right)+T\left(i,j\right)\right]+q_{0}\times 3^{\left(0.1T-3.7\right)}\right\}$$

(6) $$\frac{\partial^{2}T}{\partial r^{2}}=\frac{T\left(i,j+1\right)-2T\left(i,j\right)+T\left(i,j-1\right)}{\rho_{t}\cdot c_{t}\cdot {\left(\mathrm{\Delta r}\right)}^{2}}=\frac{1}{\rho_{t}\cdot c_{t}\cdot {\left(\mathrm{\Delta r}\right)}^{2}}\left\{\frac{\lambda_{t}}{r^{2}}\cdot \frac{\partial }{\partial r}\cdot \left[r^{2}\cdot \frac{\partial T\left(i,j+1\right)-2\partial T\left(i,j\right)+\partial T\left(i,j-1\right)}{\partial r}\right]+\rho_{b}\cdot c_{b}\cdot \omega \cdot \left[T_{a}-T\left(i,j+1\right)+2T\left(i,j\right)-T\left(i,j-1\right)\right]+q_{0}\times 3^{\left(0.1T-3.7\right)}\right\}$$

The boundary and initial conditions become:

(7) $$t=0\;s,r=0\;\mathrm{cm},T=37^{{}^{\circ}}C$$

(8) $$r=0\;\mathrm{cm},T=37^{{}^{\circ}}C$$

(9) $$r=\left(0\;\mathrm{cm},10\;\mathrm{cm}\right)$$

All data analysis and numerical simulations were performed in Matlab (Natick, MA). For the temperature calculation, Table 1 presents an overview of all fixed parameters and their values from references ^20^, ^27^.

**TABLE 1:** The partial physiological parameters of the human body

Parameter	Symbol	Value
Blood density	$\rho_{b}$	$1050\;\mathrm{kg}/m^{3}$
Blood‐specific heat capacity	$c_{b}$	3800J/Kg⋅K
Blood thermal conductivity	$\lambda_{b}$	0.5W/m⋅K
Tissue density	$\rho_{t}$	$1030\;\mathrm{kg}/m^{3}$
Tissue‐specific heat capacity	*c*_t_	3700J/Kg⋅K
Tissue thermal conductivity	*λ*_t_	0.49W/m⋅K
Perfusion rate of gray matter	$\omega_{\text{gray}}$	80ml/min⋅100g
Perfusion rate of white matter	$\omega_{\text{white}}$	20ml/min⋅100g
Metabolic rate of gray matter	$q_{\text{gray}}$	$16,700\;W/m^{3}$
Metabolic rate of white matter	$q_{\text{white}}$	$4175\;W/m^{3}$

### 2.3 Experimental study on in vitro simulation

**FIGURE 2:**
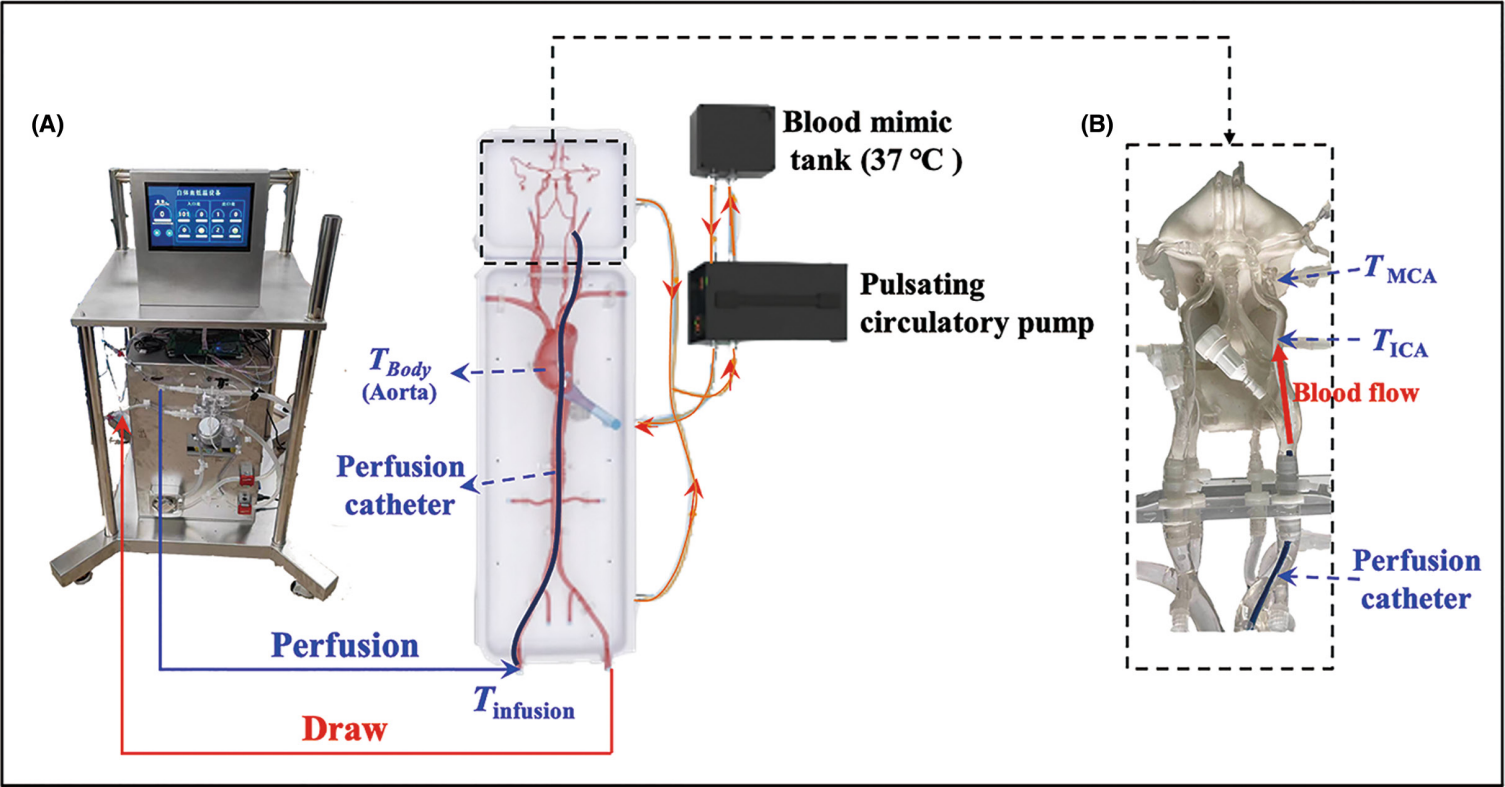
(A) The cerebral vascular model included 3D‐printing blood arteries, a blood mimic sink, and a pulsing circulatory pump; (B) temperatures (*T*_infusion_, *T*_ICA_, and *T*_MCA_) were evaluated inside the in vitro experiment

In order to obtain the blood temperature $T_{a}\left({}^{{}^{\circ}}C\right)$ of the perfusion by the IA‐SCAI prototype instrument system, experiments were carried out on a 1:1 3D‐printed physical cerebral vascular model (Preclinic Medical, Shanghai, China) which was filled with simulated blood (56 % glycerin and 44 % bi‐distilled water,^28^ 37°C). (Figure 2) A pulsing pump (pulsation frequency: 80 beats/min, flow range of the pump: 0–1000 ml/min) and a constant temperature (37°C) water tank powered the circulatory in the cerebral vascular model. To acquire the flow of simulated blood within the carotid artery (CCA), we may change the flow of this circulatory pump (injected into the aortic arch, as illustrated in Figure 2A). We employed an interventional catheter (Codman ENVOY® Catheter, 5F) to simulate the IA‐SCAI pathway in the cerebral vascular model with the prototype instrument system in vitro.

A thermocouple K‐type multichannel temperature tester (AT4204; Applent) was used to monitor the cerebral vascular temperatures in different positions of the model (cold autologous blood inflow temperature at the femoral (*T*_infusion_), temperature at ICA (*T*_ICA_), and MCA (*T*_MCA_)). The MCA segment was thought to be the site of cerebral ischemia infarctions. The temperature in the aorta can be equivalent to the core temperature of the human body (*T*_*Body*_) during the IA‐SCAI process. The K‐type thermocouple was placed into the cerebral vascular model (Figure 2B) through a hemostatic valve and calibrated to 0.1°C throughout a temperature range of −50.0 to 250.0°C. The room temperature during the in vitro experiment was about 20.0°C, whereas the temperature of the cerebral vascular model was kept at 36.3 ± 0.1°C, and the cooling water tank temperature was set at 5.0 ± 0.2°C. The CCA flow inside the in vitro experiment was set to 180 ml/min to conform with the parameters of cerebral blood flow in patients with AIS because blood perfusion in the developing infarct (ischemic penumbra) was lowered to 40%–50% (based on normal 400 ml/min).^20^, ^29^, ^30^

## 3 RESULTS

**FIGURE 3:**
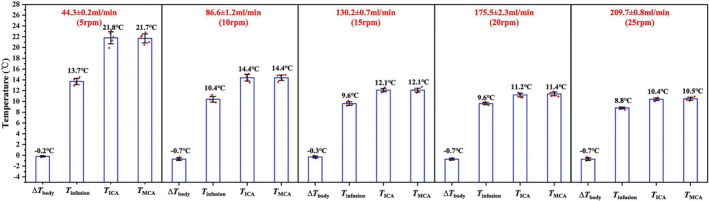
*T*_infusion_, *T*_ICA_, *T*_MCA_, and Δ*T*_*body*_ temperatures at various rotation speeds of the blood pump (5 rpm, 10 rpm, 15 rpm, 20 rpm, and 25 rpm correspond to 44.3 ± 0.2 ml/min (flow1), 86.6 ± 1.2 ml/min (flow2), 130.2 ± 0.7 ml/min (flow3), 175.5 ± 2.3 ml/min (flow4), and 209.7 ± 0.8 ml/min (flow5), respectively)

Using the prototype instrument system, Figure 3 summarizes the temperatures of autologous blood at the input of the femoral artery (*T*_infusion_), ICA (*T*_ICA_), MCA (*T*_MCA_), and the change in the core body temperature (Δ*T*_Body_) of the cerebral vascular model at varied flow rates. These data were utilized to calculate and analyze the brain tissue temperature for the IA‐SCAI. Higher flow rates resulted in lower temperatures of autologous blood, and the core body temperature did not alter significantly (−0.7 ~ +0.2°C) after 1‐h perfusion. Hypothermia is generally accepted that a core body temperature of <35°C. This can then be further subdivided into mild (32 ~ 35°C), moderate (28 ~ 32°C), and severe (<28°C) hypothermia.^31^ Through the prototype instrument, changing the volume of perfusion and the heat transfer efficiency of the cooling water tank could accomplish different targeted cerebral vascular temperatures. To keep the *T*_MCA_ temperature between 32°C and 35°C, the rotation speed can be reduced to 2 ~ 3 rpm or the cooling tank temperature set to 9 ~ 10°C. *T*_infusion_ dropped from 13.7 to 8.8°C and *T*_ICA_ dropped from 21.8 to 10.4 as the perfusion flow rose from 44.3 ml/min to 209.7 ml/min, and the temperature change of *T*_MCA_ was similar to *T*_ICA_. Due to the inverse relationship between total heat transfer and flow rate, heat transmission decreases as flow rate increases. All different hyperthermia (mild, moderate, and severe) could be achieved due to the adequate cooling capacity of the prototype with the lowest cooling temperature into the blood vessel of 10.5°C at 25 rpm (209.7 ± 0.8 ml/min).

**FIGURE 4:**
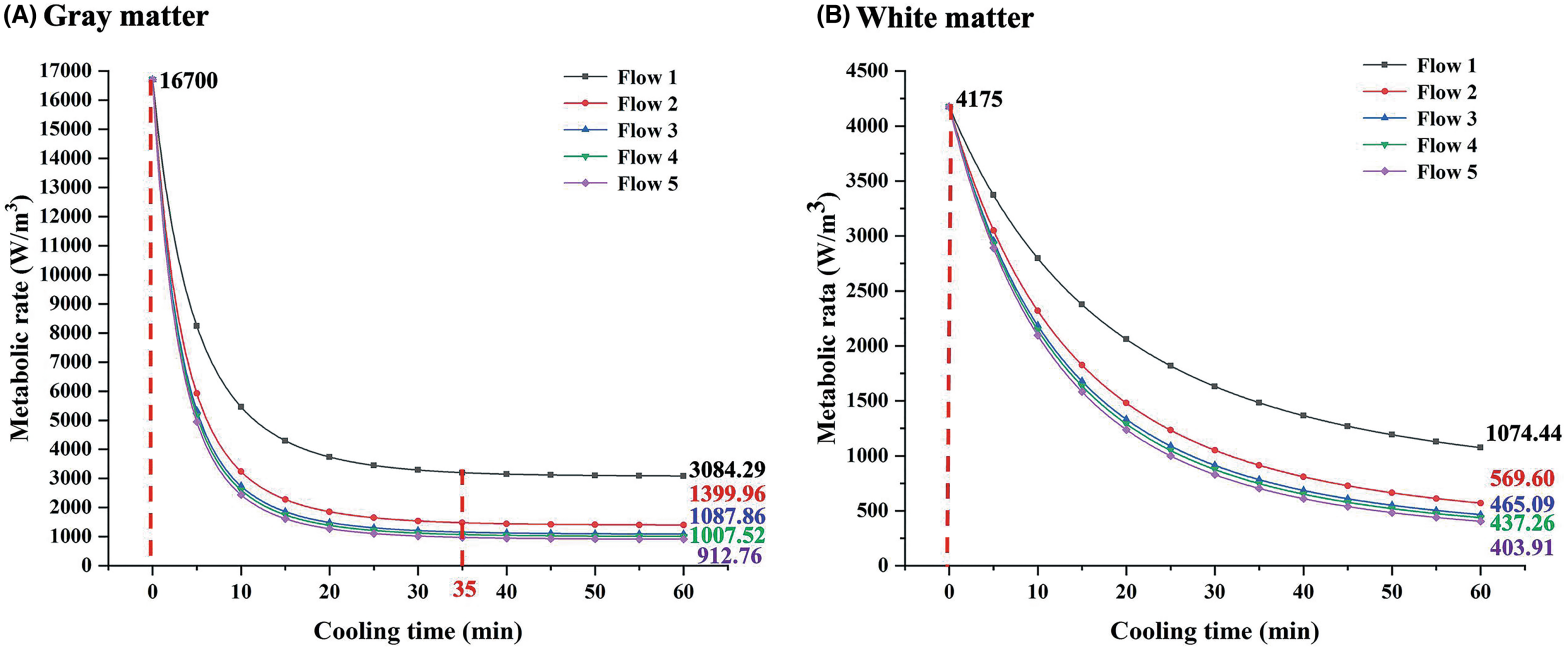
Effect of autologous blood infusion rates on the metabolic rate of the GM (A) and white (B) WM of the brain

In addition to heat conduction between blood and brain tissue,^31^, ^32^, ^33^ metabolic heat production of white matter (WM) and gray matter (GM) has effects on brain temperature.^34^ Specifically, neuron‐rich GM requires 2.5 times more ATP than WM,^35^ and the baseline metabolic rate of the GM and WM of the brain at 37°C is 16,700 W/m^3^ and 4175 W/m,^3^ respectively.^20^ Figure 4(a‐b) shows the influence of different autologous blood infusion rates on the metabolic rate of the GM and WM. As shown in Figure 4(a‐b), high infusion rates resulted in a faster metabolic rate, and the metabolic rate under the condition of the highest flow (209.7 ± 0.8 ml/min) were reached to 912.76 W/m^3^ for GM and 403.91 W/m^3^ for WM within 1 h, respectively. The metabolic rate of GM decreased quicker than WM. After 35 minutes of cooling, the gray matter achieved its low stable metabolic rate, whereas the white matter continued to decline after 1 hour of cooling.

**FIGURE 5:**
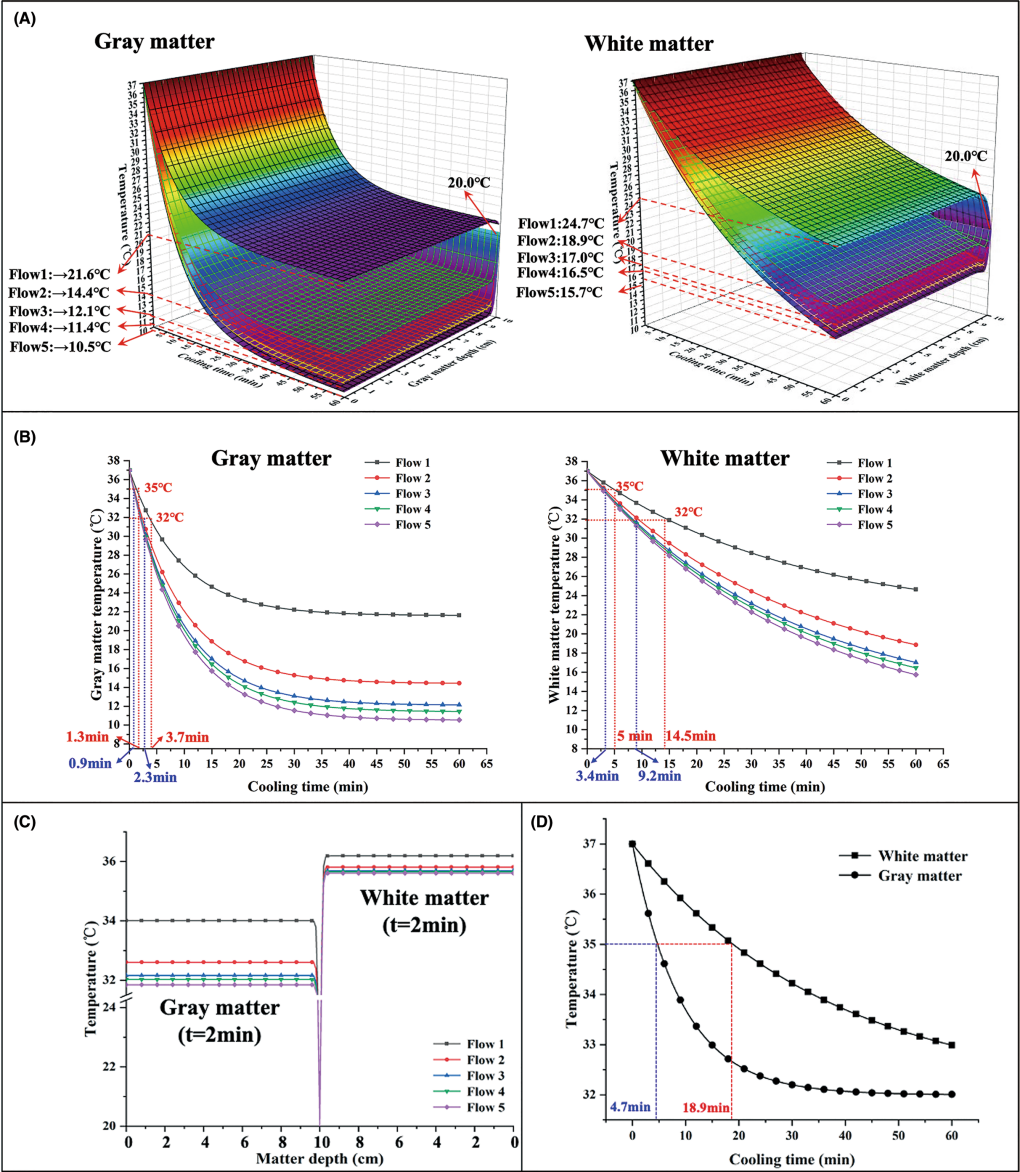
(A) Three‐dimensional diagram representing the combined effect of cooling time and depth on temperature. (B) Effect of autologous blood infusion rates on the transient temperature of the gray and white matter of the brain. (C) Effect of autologous blood infusion rates on the steady‐state temperature of the gray and white matter of the brain at 2 min. (D) Effect of autologous blood infusion rates on the transient temperature of the gray and white matter of the brain (*T*_MCA_ = 32°C)

The combined impact of cooling duration infusion (within 60 min) and brain tissue depth (within 10 cm) on the temperature reduction of the brain tissue is shown in Figure 5A. Faster infusion rates resulted in quicker hypothermia onset and lower brain temperatures. After a 60‐min cooling infusion, the temperature of GM eventually tended to be stable at MCA (*T*_MCA_), whereas the temperature of the WM continued to decline. Compared with WM, the temperature change of GM was more obvious under different perfusion flow, for example, an infusion rate of 209.7 ± 0.8 ml/min (flow 5) resulted in a minimum temperature of 10.5°C at the GM. Between flow 1 and flow 5, the transient temperature of GM was 3–5°C greater than that of WM. Mild‐to‐moderate hypothermia (35°C) was established in the GM within 1.3 min, which was faster than the WM (within 5 min) for the autologous blood infusion rate of flow 1, as shown in Figure 5B. As the flow increased, it took less time to reach the hypothermia temperature and had a shorter period of mild hypothermia (32–35°C) on the brain tissue, resulting in GM having a shorter duration (2.4 min by flow 1) of mild hypothermia than WM (9.5 min by flow 1).

The temperature distribution after 60 minutes of autologous blood is also shown in Figure 5A. The temperature of the brain fluctuated little under steady‐state circumstances, but near the outer boundary of the brain, the temperature started to approach 20°C (the boundary temperature). *T*_MCA_ = 32°C could be used to induce long‐term hypothermia perfusion of autologous blood, as demonstrated in Figure 5c‐d, where gray matter reached mild‐to‐moderate hypothermia 14.2 min before white matter.

## 4 DISCUSSION

We present the first experimental prototype instrument system which permits continuous intra‐arterial autogenous blood perfusion for safely and effectively selective cooling of AIS. To evaluate how the efficiency of the prototype system cooled brain tissue, this study used an in vitro simulation loop device and a computational numerical theoretical model to explore the effect of different flow rates in terms of times (*t*) and depths (*r*) on the brain tissue temperature during the IA‐SCAI. The prototype instrument system could attain target brain tissue temperatures ranging from 10.5 to 35°C for a long duration by using varied infusion flows inside the in vitro simulation loop device, which was based on a 1:1 3D‐printed physical cerebrovascular model using actual CT data. The development of an IA‐SCAI prototype instrument system is critical to achieving clinical hypothermia transition. It serves as a research tool for determining the optimal parameters for autologous blood selective hypothermia and confirming the mechanism of neuroprotective effect for hypothermia. In this study, an in vitro experimental platform was fabricated based on the real human intracranial vascular system, which provides a convenient method to evaluate the feasibility and cooling efficiency of the IA‐SCAI prototype instrument. Konstas demonstrated that the method including the in vitro study and a numerical theoretical model was an attempt to determine the feasibility of selective brain cooling with intracarotid saline infusion.^20^ Our group also used this method to conduct a feasibility assessment on the intravascular interventional catheter^36^ and heat exchanger^37^ in the IA‐SCAI prototype system.

### 4.1 Comparisons with different hypothermic therapies

Currently, hypothermic therapy can be achieved using a variety of cooling methods and procedures, and there are the three most critical considerations: cooling targeting, cooling rate, and side effects (Table 2). Surface cooling is the common traditional hypothermic therapy. However, this approach was difficult to achieve targeted cooling on brain tissue with a poor cooling rate (only 3.5°C/h).^38^ Nasal cooling and intravascular non‐perfusion device cooling as novel brain cooling have evolved into a viable and promising neuroprotective hypothermic therapy but with an undesirable phenomenon of a drop (1.1 ~ 3°C)^39^, ^40^ in the core temperature to cause systemic hypothermia, which can lead to adverse effects outlined in Table 2. In 2002, our group proposed intra‐arterial selective cooling saline infusion as an SBC approach to achieve targeted cooling on brain tissue.^41^ It has since been widely employed in rodent animal models,^12^, ^42^ large animal models,^40^, ^43^, ^44^ and stroke patients^14^, ^15^, ^45^ with the safety and feasibility. However, an excessive amount of saline perfusion into intracranial brain tissue might increase the strain on the heart, lower the hematocrit, and limit the duration of hypothermia for AIS patients.^8^

**TABLE 2:** The comparison of different hypothermic therapies

Hypothermic therapies	Companies and systems	Selective brain cooling	Cooling rate	Side effects
Surface cooling	Emcools: Flexipad^38^ Natus Medical: Olympic cool‐cap^46^ Cryothermic systems: Cooling pack C.R.Bard: Artic Sun^38^	No	2 ~ 6°C/h	Easy to cause systemic hypothermia^8^: shivering, infection, effects on drug clearance, hyperglycemia, electrolyte imbalance, effects on the cardiovascular system, changes in urine output, and effects on coagulation.
Nasal cooling	Bene Chill: Rhino chill^47^	No	5.2 ± 1.9°C/h^47^	Reduction in core temperature (1.1 ~ 2.2°C).^39^
Intravascular non‐perfusion device cooling	Pforzheim: Acandis^40^ Zoll: Thermoguard XP system^38^	Yes	1°C per 8.5 min^40^	Reduction in core temperature (~3°C).^40^
Intravascular perfusion saline cooling	FocalCool: Seiratherm^43^	Yes	2.2 ± 2.5°C/ min^43^	Saline load increased the burden on the heart and reduce the hematocrit.^43^

In 2009, our group devised the intra‐arterial selective cooling autogenous blood infusion (IA‐SCAI) in answer to the problem of prolonging ongoing treatments of selective brain hypothermia in AIS patients without the abovementioned negative effects.^17^ In the study, the IA‐SCAI prototype instrument system had a little change in the core temperature (−0.7 ~ +0.2°C) through the in vitro experiment and ensured long‐term hypothermia maintenance for 1 h.

### 4.2 Mathematical models for estimation of brain temperature for in vivo data

#### 4.2.1 Pre‐clinical experiments

For rodent models, some studies have concluded that selective brain cooling treatment immediately after ischemic stroke significantly improved neurobehavioral function. These findings are beneficial to unveil the underlining mechanisms involved in neuroprotection against acute cerebral ischemic injuries.^48^, ^49^ However, in some of the large animal and non‐human primate studies, the reduction of infarct size and improved functional outcomes induced by selective brain cooling were not statistically different from their control groups, which may ascribe to the small sample size. Therefore, a larger number of subjects might be required to demonstrate the efficacy of this technique.^50^, ^51^

The time to target temperature and the brain temperature from the in vivo and in vitro study is compared in Table 3. The calculation results from the numerical theoretical model in this manuscript were compatible with the brain temperature estimation of rat, monkey, and human during IA‐SCAI. For the rat, the predicted values calculated by this model were not significantly different from those obtained in vivo, which can achieve rapid cooling (35°C) on brain temperature within 10 min. The numerical analysis of brain temperature obtained by the monkey, and the time to reach the target temperature was slower than that of in vivo experiments. For comparison with human data, the time to target temperature (35°C) in the numerical model was 10.4 min which was slower than in the in vivo study.

**TABLE 3:** The time to target temperature and brain temperature between the in vivo and in vitro study

Subject	The in vivo study				The in vitro study			
	Infusion rate	Infusion duration	Time to target temp	Brain temp	Infusion rate	Infusion duration	Time to target temp	Brain temp
Rat	0.6 ml/min	10 min	<10 min	<35°C	0.6 ml/min	60 min	3.4 min	35°C
Monkey	5 ml/min	20 min	10 min	34°C	5 ml/min	60 min	14.8 min	34°C
Human^a^	10‐30 ml/min	5‐10 min	Not mentioned	35°C	10‐30 ml/min	60 min	10.4 min	35°C

^a^The in vivo study was chosen from the following reference: rat,^52^ monkey,^53^ and human.^14^

The baseline cerebral blood flow (*ω*_0_) and metabolic rate (*q*_*0*_) were used for the calculation by the numerical theoretical model in this study (Table 4). The mean cerebral blood flow (*ω*_0_) of rat, monkey, and human was 114 ml/(min.100 g), 46.12 ml/(min.100 g), and 36.56 ml/(min.100 g), respectively. And, the metabolic rate (*q*_*0*_) was 5124.25 W/m^3^, 7210 W/m^3^, and 16,700 W/m^3^, respectively.

**TABLE 4:** The cerebral blood flow (*ω*_0_) and metabolic rate (*q*_*0*_) in rat, monkey, and human

Subject	Temp	Cerebral blood flow (*ω*_0_)	Ref.
Rat	38°C	108 ml/(min.100 g)	Hagerdal et al.^54^
	37.5°C	113 ml/(min.100 g)	Frietsch et al.^55^
	37.5°C	121 ml/(min.100 g)	Kraff et al.^56^
Monkey	–	46.12 ml/(min.100 g)	Daniel E et al.^57^
Human^a^	37°C	40 ml/(min.100 g)	Stone et al.^58^
	37°C	25 ml/(min.100 g)	Murkin et al.^59^
	37°C	33 ml/(min.100 g)	Stephan et al.^60^
	37°C	34 ml/(min.100 g)	Stephan et al.^60^
	38°C	50.6 ml/(min.100 g)	Konstas et al.^20^

Subject	Metabolic heat production	Metabolic rate (*q*_*0*_)	Ref.
Rat	1.99 W/400 g	5124.25 W/m^3^	Refinetti et al.^61^
Monkey	7 W/kg	7210 W/m^3^	Adair et al.^62^
Human^a^	–	16,700 W/m^3^	Konstas et al.^20^

^a^The mass density was set as 1030 kg/m^3^.^20^

#### 4.2.2 Clinical data

Theoretically, controlled flow rates of autologous arterial blood to perfuse into the brain, the human body's blood circulation should be unaffected， which enable normal cerebral blood flow to be restored after the vessel recanalized, and the period of intravascular hypothermic perfusion could be lengthened significantly without hemodilution. Therefore, in the mathematical model for IA‐SCAI compared with IA‐SCI saline perfusion, we were able to enhance the blood flow of autologous blood perfusion and improve the cooling capacity of IA‐SCAI, allowing it to reach mild‐to‐moderate hypothermia faster and last longer time (within 5 minutes by >40 ml/min, as shown in Figure 5).

According to Konstas's model for brain temperature estimate of IA‐SCI by saline (30 ml/min by 60 min), mild‐to‐moderate hypothermia was achieved within 10 minutes after the infusion, which was 18–42 times quicker than noninvasive whole‐body cooling (3–7 hours) and 10–20 times faster than whole‐body endovascular cooling (10–20 hours).^63^, ^64^ Despite the fact that IA‐SCI saline perfusion could provide targeted brain tissue cooling without influencing the human body's core temperature, the burden on the heart and hematocrit limited its cooling duration.^65^ The hematocrit was reduced by 31% and 25% after a 60‐minute saline infusion at 30 and 50 ml/min. The mild‐to‐moderate hypothermia may be safely sustained for 90 minutes at a rate of 50 ml/min and 180 minutes at a rate of 30 ml/min.^20^ In order to provide efficient neuroprotective effects, hypothermia treatment in AIS patients should be maintained for a long time, which meant that IA‐SCI saline perfusion was not acceptable for longer time usage. And it's challenging to keep brain temperature below 35°C with lower saline infusion rates.^66^

As shown in Table 5, Choi et al.^45^ selectively infused 33 ml/min cold saline (4–17°C) into 18 non‐stroke patients for 10 min. The target brain temperature decreased by an average of 0.84°C whereas the core body temperature dropped by 0.15°C. The same studies of IA‐CSI were also conducted by Chen^14^ and Wu^15^ through the infusion of 10‐30 ml/min of 4°C saline on AIS patients. They also achieved a 2°C drop in brain temperature and maintained a core body temperature change between 0.1°C and 0.5°C. We circulated the simulated cold blood (13.7°C) with a higher infusion rate (44.3 ± 0.2 ml/min) for 60 min to achieve a lower brain temperature drop (−4.3°C). For comparison, the time to the target temperature in this study was similar to Chio's results (within 10 minutes) with little changes in core temperature. During the in vitro experiment, the brain temperature drop was bigger due to the use of a higher perfusion flow.

**TABLE 5:** Summary of clinical studies on intra‐arterial cold saline infusion

Authors	Subject	Infusion	Infusion rate	Infusion duration	Time to target temp	Brain temp	Core body temp
Choi et al., 2010^45^	Non‐stroke patients	Saline 4–17°C	33 ml/min	10 min	<10 min	−0.84°C	−0.15°C
Chen et al., 2016^14^	AIS patients	Saline 4°C	10‐30 ml/min	5‐10 min	Not mentioned	−2°C	−0.1°C
Wu et al., 2018^15^	AIS patients	Saline 4°C	10‐30 ml/min	5‐10 min	Not mentioned	Not mentioned	−0.5°C
This in vitro study	–	Simulated auto‐blood 13.7°C	44.3 ± 0.2 ml/min	60 min	3.7 min (Gray matter)	−4.3°C	−0.2°C

### 4.3 Limitations and prospective of the study

Despite positive results in rodent and small mammal research, the neuroprotective effect of IA‐SCAI on AIS patients has been still debatable and cannot be widely used in clinical practice. The absence of rapid, precise, safe, and effective IA‐SCAI hypothermia brain protection equipment, as well as the lack of accepted clinical pathway of hypothermia therapy for AIS patients, limits the clinical promotion of this technology. As a result, the IA‐SCAI prototype system described in this study may be used as a research tool to solve these problems.

Next, we aim to construct a more realistic in vitro cerebrovascular and tissue simulation system based on the complex anatomy of the brain and continue to evaluate the effect of IA‐SCAI in combination with Pennes' complete bio‐thermal mathematical equation. To increase the safety of autologous blood perfusion by the IA‐SCAI system for large animal models, the rolling blood pump described in this study should be replaced with a centrifugal blood pump in order to ensure long‐term safe blood extracorporeal circulation as it has a lower hemolysis rate,^67^ and the anticoagulant coating should be applied onto tubes and equipment.^68^ In the future investigation, we will perform an in vivo study of IA‐SCAI to evaluate the safety and efficacy of the prototype instrument system by using large AIS animal models. Our next step is to apply the prototype to the research of large animals to prepare for clinical validation, which needs to focus on the accurate temperature control of autologous blood cooling, the steady perfusion of intravascular autologous blood flow, and the preparation of AIS animal models.

## 5 CONCLUSION

The prototype instrument system of IA‐SCAI developed in this study offers a preliminary safety and efficacy assessment through the mathematical brain temperature estimation and the in vitro experiment. The experimental results showed that: (1) this prototype instrument system could safely cool simulated blood in vitro and return it to the target cerebral blood vessel. Different hyperthermia (mild, moderate, and severe) could be achieved by the system due to the adequate cooling capacity of the prototype, which could provide the lowest cooling temperature into the blood vessel of 10.5°C at 25 rpm (209.7 ± 0.8 ml/min). And, the core body temperature did not alter significantly (−0.7 ~ +0.2°C) after 1‐h perfusion. (2) Using a mathematical model of biological heat transfer, the cooling rate and temperature distributions of the brain tissue were analyzed during the IA‐SCAI, which showed a 2°C decrease within the initial 5 min infusion by 44 ml/min and 13.7°C perfusate. This system is a reliable tool for neuroprotective studies on stroke animal models. This technique can circumvent the adverse effects of saline perfusion and promote the clinical application of an autologous blood perfusion system for stroke therapy.

## CONFLICT OF INTEREST

All authors declare that they have no conflict of interest.

## Data Availability

The data supporting the findings of this study are available from the corresponding author upon reasonable request. They are not publicly available due to ethical restriction.

## References

[1] Collaborators GBDLRoS , Feigin VL , Nguyen G , et al. Global, regional, and country‐specific lifetime risks of stroke 1990 and 2016. N Engl J Med. 2018;379(25):2429‐2437. 30575491 10.1056/NEJMoa1804492 PMC6247346

[2] Krishnamurthi RV , Feigin VL , Forouzanfar MH , et al. Global and regional burden of first‐ever ischaemic and haemorrhagic stroke during 1990–2010: findings from the global burden of disease study 2010. Lancet Glob Health. 2013;1(5):e259‐e281. 25104492 10.1016/S2214-109X(13)70089-5 PMC4181351

[3] Powers WJ , Rabinstein AA , Ackerson T , et al. Guidelines for the early management of patients with acute ischemic stroke: 2019 update to the 2018 guidelines for the early management of acute ischemic stroke: a guideline for healthcare professionals from the American Heart Association/American Stroke Association. Stroke. 2019;50(12):e344‐e418. 31662037 10.1161/STR.0000000000000211

[4] LeCouffe NE , Kappelhof M , Treurniet KM , et al. A randomized trial of intravenous alteplase before endovascular treatment for stroke. N Engl J Med. 2021;385(20):1833‐1844. 34758251 10.1056/NEJMoa2107727

[5] Goyal M , Menon BK , van Zwam WH , et al. Endovascular thrombectomy after large‐vessel ischaemic stroke: a meta‐analysis of individual patient data from five randomised trials. Lancet. 2016;387(10029):1723‐1731. 26898852 10.1016/S0140-6736(16)00163-X

[6] Wu T‐C , Grotta JC . Hypothermia for acute ischaemic stroke. Lancet Neurol. 2013;12(3):275‐284. 23415567 10.1016/S1474-4422(13)70013-9

[7] Yenari MA , Han HS . Neuroprotective mechanisms of hypothermia in brain ischaemia. Nat Rev Neurosci. 2012;13(4):267‐278. 22353781 10.1038/nrn3174

[8] Wu L , Wu D , Yang T , et al. Hypothermic neuroprotection against acute ischemic stroke: the 2019 update. J Cereb Blood Flow Metab. 2020;40(3):461‐481. 31856639 10.1177/0271678X19894869 PMC7026854

[9] Piironen K , Tiainen M , Mustanoja S , et al. Mild hypothermia after intravenous thrombolysis in patients with acute stroke: a randomized controlled trial. Stroke. 2014;45(2):486‐491. 24436240 10.1161/STROKEAHA.113.003180

[10] Wu L , Huber M , Wu D , et al. Intra‐arterial cold saline infusion in stroke: historical evolution and future prospects. Aging Dis. 2020;11(6):1527‐1536. 33269105 10.14336/AD.2020.0325 PMC7673854

[11] Chen J , Fredrickson V , Ding Y , et al. Enhanced neuroprotection by local intra‐arterial infusion of human albumin solution and local hypothermia. Stroke. 2013;44(1):260‐262. 23192754 10.1161/STROKEAHA.112.675462

[12] Ding Y , Li J , Luan X , et al. Local saline infusion into ischemic territory induces regional brain cooling and neuroprotection in rats with transient middle cerebral artery occlusion. Neurosurgery. 2004;54(4):956‐964. discussion 964–955, 965. 15046664 10.1227/01.neu.0000114513.96704.29

[13] Wu D , Chen J , Hussain M , et al. Selective intra‐arterial brain cooling improves long‐term outcomes in a non‐human primate model of embolic stroke: efficacy depending on reperfusion status. J Cereb Blood Flow Metab. 2020;40(7):1415‐1426. 32126876 10.1177/0271678X20903697 PMC7308521

[14] Chen J , Liu L , Zhang H , et al. Endovascular hypothermia in acute ischemic stroke: pilot study of selective intra‐arterial cold saline infusion. Stroke. 2016;47(7):1933‐1935. 27197848 10.1161/STROKEAHA.116.012727 PMC4927369

[15] Wu C , Zhao W , An H , et al. Safety, feasibility, and potential efficacy of intraarterial selective cooling infusion for stroke patients treated with mechanical thrombectomy. J Cereb Blood Flow Metab. 2018;38(12):2251‐2260. 30019993 10.1177/0271678X18790139 PMC6282221

[16] Mattingly TK , Lownie SP . Cold blood perfusion for selective hypothermia in acute ischemic stroke. Brain Circ. 2019;5(4):187‐194. 31950094 10.4103/bc.bc_17_19 PMC6950509

[17] Cheng H , Ji X , Ding Y , et al. Focal perfusion of circulating cooled blood reduces the infarction volume and improves neurological outcome in middle cerebral artery occlusion. Neurol Res. 2009;31(4):340‐345. 19508815 10.1179/174313209X443982

[18] Pennes HH . Analysis of tissue and arterial blood temperatures in the resting human forearm. J Appl Physiol. 1948;85(1):5‐34. 10.1152/jappl.1998.85.1.5 9714612

[19] Bommadevara M , Zhu LJB . Temperature difference between the body core and arterial blood supplied to the brain during hyperthermia or hypothermia in humans. Biomech Model Mechanobiol. 2002;1(2):137‐149. 14595546 10.1007/s10237-002-0011-2

[20] Konstas A‐A , Neimark MA , Laine AF , Pile‐Spellman J . A theoretical model of selective cooling using intracarotid cold saline infusion in the human brain. J Appl Physiol. 2007;102(4):1329‐1340. 17170208 10.1152/japplphysiol.00805.2006

[21] Li H , Chen RK , Tang Y , Meurer W , Shih AJ . An experimental study and finite element modeling of head and neck cooling for brain hypothermia. J Therm Biol. 2018;71:99‐111. 29301706 10.1016/j.jtherbio.2017.10.022

[22] Lutz Y , Daschner R , Krames L , et al. Modeling selective therapeutic hypothermia in case of acute ischemic stroke using a 1D hemodynamics model and a simplified brain geometry. Math Biosci Eng. 2019;17(2):1147‐1167. 32233574 10.3934/mbe.2020060

[23] Utsuki T . Automatic control system of brain temperature by air‐surface cooling for therapeutic hypothermia. Annu Int Conf IEEE Eng Med Biol Soc. 2013;2013:3741‐3744. 24110544 10.1109/EMBC.2013.6610357

[24] Yin L , Jiang H , Zhao W , Li HJM . Inducing therapeutic hypothermia via selective brain cooling: a finite element modeling analysis. Med Biol Eng Comput. 2019;57(6):1313‐1322. 30756230 10.1007/s11517-019-01962-7

[25] Michenfelder JD , Milde JH . The relationship among canine brain temperature, metabolism, and function during hypothermia. Anesthesiology. 1991;75(1):130‐136. 2064037 10.1097/00000542-199107000-00021

[26] Xu X , Tikuisis P , Giesbrecht G . A mathematical model for human brain cooling during cold‐water near‐drowning. J Appl Physiol. 1999;86(1):265‐272. 9887139 10.1152/jappl.1999.86.1.265

[27] Diao C , Zhu L , Wang H . Cooling and rewarming for brain ischemia or injury: theoretical analysis. Ann Biomed Eng. 2003;31(3):346‐353. 12680732 10.1114/1.1554924

[28] Cattaneo G , Schumacher M , Wolfertz J , Jost T , Meckel S . Combined selective cerebral hypothermia and mechanical artery recanalization in acute ischemic stroke: in vitro study of cooling performance. AJNR Am J Neuroradiol. 2015;36(11):2114‐2120. 26251430 10.3174/ajnr.A4434 PMC7964854

[29] Kaneko N , Komuro Y , Yokota H , Tateshima S . Stent retrievers with segmented design improve the efficacy of thrombectomy in tortuous vessels. J Neurointerv Surg. 2019;11(2):119‐122. 30045949 10.1136/neurintsurg-2018-014061

[30] Shimosegawa E , Hatazawa J , Ibaraki M , Toyoshima H , Suzuki A . Metabolic penumbra of acute brain infarction: a correlation with infarct growth. Ann Neurol. 2005;57(4):495‐504. 15786459 10.1002/ana.20427

[31] Faulds M , Meekings T . Temperature management in critically ill patients. Cont Educ Anaesthesia Crit Care Pain. 2013;13(3):75‐79.

[32] Ko TS , Mavroudis CD , Baker WB , et al. Non‐invasive optical neuromonitoring of the temperature‐dependence of cerebral oxygen metabolism during deep hypothermic cardiopulmonary bypass in neonatal swine. J Cereb Blood Flow Metab. 2020;40(1):187‐203. 30375917 10.1177/0271678X18809828 PMC6928559

[33] Yannick L , Rosa D , Krames L , et al. Estimating local therapeutic hypothermia in case of ischemic stroke using a 1D hemodynamics model and an energetic temperature model. Annu Int Conf IEEE Eng Med Biol Soc. 2019;2019(9):3983‐3986. 31946744 10.1109/EMBC.2019.8856447

[34] Iguchi A , Haneda K , Sato S , Horiuchi T . Determination of safe interval of circulatory arrest from the cerebral metabolic aspect. Tohoku J Exp Med. 1986;149(2):191‐204. 3750321 10.1620/tjem.149.191

[35] Kaiser EE , West FD . Large animal ischemic stroke models: replicating human stroke pathophysiology. Neural Regen Res. 2020;15(8):1377‐1387. 31997796 10.4103/1673-5374.274324 PMC7059570

[36] Jiang M , Li M , Gao Y , et al. Design and evaluation of an air‐insulated catheter for intra‐arterial selective cooling infusion from numerical simulation and in vitro experiment. Med Eng Phys. 2022;99:103736. 35058029 10.1016/j.medengphy.2021.103736

[37] Jiang M , Gao Y , Wu C , et al. The blood heat exchanger in intra‐arterial selective cooling infusion for acute ischemic stroke: a computational fluid‐thermodynamics performance, experimental assessment and evaluation on the brain temperature. Comput Biol Med. 2022;145:105497. 35398811 10.1016/j.compbiomed.2022.105497

[38] Vaity C , Al‐Subaie N , Cecconi M . Cooling techniques for targeted temperature management post‐cardiac arrest. Crit Care. 2015;19:103. 25886948 10.1186/s13054-015-0804-1 PMC4361155

[39] Abou‐Chebl A , Sung G , Barbut D , Torbey M . Local brain temperature reduction through intranasal cooling with the RhinoChill device: preliminary safety data in brain‐injured patients. Stroke. 2011;42(8):2164‐2169. 21680904 10.1161/STROKEAHA.110.613000

[40] Cattaneo G , Schumacher M , Maurer C , et al. Endovascular cooling catheter for selective brain hypothermia: an animal feasibility study of cooling performance. AJNR Am J Neuroradiol. 2016;37(5):885‐891. 26705319 10.3174/ajnr.A4625 PMC7960302

[41] Ding Y , Li J , Rafols JA , Phillis JW , Diaz FG . Prereperfusion saline infusion into ischemic territory reduces inflammatory injury after transient middle cerebral artery occlusion in rats. Stroke. 2002;33(10):2492‐2498. 12364743 10.1161/01.str.0000028237.15541.cc

[42] Ding YH , Li J , Rafols JA , Ding Y . Reduced brain edema and matrix metalloproteinase (MMP) expression by pre‐reperfusion infusion into ischemic territory in rat. Neurosci Lett. 2004;372(1–2):35‐39. 15531084 10.1016/j.neulet.2004.09.010

[43] Caroff J , King RM , Mitchell JE , et al. Focal cooling of brain parenchyma in a transient large vessel occlusion model: proof‐of‐concept. J Neurointerv Surg. 2020;12(2):209‐213. 31363042 10.1136/neurintsurg-2019-015179

[44] Mattingly TK , Denning LM , Siroen KL , et al. Catheter based selective hypothermia reduces stroke volume during focal cerebral ischemia in swine. J Neurointerv Surg. 2016;8(4):418‐422. 25676148 10.1136/neurintsurg-2014-011562

[45] Choi JH , Marshall RS , Neimark MA , et al. Selective brain cooling with endovascular intracarotid infusion of cold saline: a pilot feasibility study. AJNR Am J Neuroradiol. 2010;31(5):928‐934. 20053807 10.3174/ajnr.A1961 PMC7964178

[46] Walas W , Bandola D , Ostrowski Z , et al. Theoretical basis for the use of non‐invasive thermal measurements to assess the brain injury in newborns undergoing therapeutic hypothermia. Sci Rep. 2020;10(1):22167. 33335145 10.1038/s41598-020-79009-3 PMC7747633

[47] Fazel Bakhsheshi M , Keenliside L , Lee TY . A novel selective cooling system for the brain: feasibility study in rabbits vs piglets. Intensive Care Med Exp. 2018;6(1):45. 30387029 10.1186/s40635-018-0211-4 PMC6212374

[48] Liu LQ , Liu XR , Zhao JY , et al. Brain‐selective mild hypothermia promotes long‐term white matter integrity after ischemic stroke in mice. CNS Neurosci Ther. 2018;24(12):1275‐1285. 30295998 10.1111/cns.13061 PMC6489965

[49] Zhao J , Mu H , Liu L , et al. Transient selective brain cooling confers neurovascular and functional protection from acute to chronic stages of ischemia/reperfusion brain injury. J Cereb Blood Flow Metab. 2019;39(7):1215‐1231. 30334662 10.1177/0271678X18808174 PMC6668511

[50] Cattaneo GF , Herrmann AM , Eiden SA , et al. Selective intra‐carotid blood cooling in acute ischemic stroke: a safety and feasibility study in an ovine stroke model. J Cereb Blood Flow Metab. 2021;41(11):3097‐3110. 34159825 10.1177/0271678X211024952 PMC8756475

[51] Wood TR , Vu PT , Comstock BA , et al. Cytokine and chemokine responses to injury and treatment in a nonhuman primate model of hypoxic‐ischemic encephalopathy treated with hypothermia and erythropoietin. J Cereb Blood Flow Metab. 2021;41(8):2054‐2066. 33554708 10.1177/0271678X21991439 PMC8327104

[52] Wu D , Shi J , Elmadhoun O , et al. Dihydrocapsaicin (DHC) enhances the hypothermia‐induced neuroprotection following ischemic stroke via PI3K/Akt regulation in rat. Brain Res. 2017;1671:18‐25. 28684048 10.1016/j.brainres.2017.06.029

[53] Wang B , Wu D , Dornbos Iii D , et al. Local cerebral hypothermia induced by selective infusion of cold lactated ringer's: a feasibility study in rhesus monkeys. Neurol Res. 2016;38(6):545‐552. 27320250 10.1080/01616412.2016.1187827

[54] Hägerdal M , Harp J , Nilsson L , Siesjöu B . The effect of induced hypothermia upon oxygen consumption in the rat brain. J Neurochem. 2010;24(2):311‐316. 10.1111/j.1471-4159.1975.tb11881.x 1113108

[55] Frietsch T , Krafft P , Piepgras A , Lenz C , Kuschinsky W , Waschke KF . Relationship between local cerebral blood flow and metabolism during mild and moderate hypothermia in rats. Anesthesiology. 2000;92(3):754‐763. 10719954 10.1097/00000542-200003000-00019

[56] Krafft P , Frietsch T , Lenz C , Piepgras A , Kuschinsky W , Waschke KF . Mild and moderate hypothermia (alpha‐stat) do not impair the coupling between local cerebral blood flow and metabolism in rats. Stroke. 2000;31(6):1393‐1400. 10835462 10.1161/01.str.31.6.1393

[57] Weiner D . Measurement of total cerebral blood flow in the monkey by external monitoring of cesium‐131. Circ Res. 1967;21(6):805‐816. 4965694 10.1161/01.res.21.6.805

[58] Donnelly C , Frobese AS , Stone HH . The effect of lowered body temperature on the cerebral hemodynamics and metabolism of man. Surg Gynecol Obstet. 1957;103(3):313‐317. 13360635

[59] Murkin JM , Farrar JK , Tweed WA , Mckenzie FN , Guiraudon G . Cerebral autoregulation and flow/metabolism coupling during cardiopulmonary bypass: the influence of PaCO_2_ . Anesth Analg. 1987;66(9):825‐832. 3113288

[60] Stephan H , Weyland A , Kazmaier S , Henze T , Menck S , Sonntag H . Acid‐base management during hypothermic cardiopulmonary bypass does not affect cerebral metabolism but does affect blood flow and neurological outcome. Br J Anaesth 1992;69(1):51‐57. 1637603 10.1093/bja/69.1.51

[61] Refinetti R . Metabolic heat production, heat loss and the circadian rhythm of body temperature in the rat. Exp Physiol. 2010;88(3):423‐429. 10.1113/eph8802521 12719767

[62] Adair ER , Adams BW . Adjustments in metabolic heat production by squirrel monkeys exposed to microwaves. J Appl Physiol Respir Environ Exerc Physiol. 1982;52(4):1049‐1058. 7085406 10.1152/jappl.1982.52.4.1049

[63] Georgiadis D , Schwarz S , Kollmar R , Schwab S . Endovascular cooling for moderate hypothermia in patients with acute stroke first results of a novel approach. Stroke. 2001;32(11):2550‐2553. 11692015 10.1161/hs1101.097382

[64] Rasmussen BH , Jorgensen HS , Reith J , Olsen TS , Weber U , Kammersgaard LP . Feasibility and safety of inducing modest hypothermia in awake patients with acute stroke through surface cooling: a case‐control study: the Copenhagen stroke study. Stroke. 2000;31(9):2251‐2256. 10978060 10.1161/01.str.31.9.2251

[65] Dietrich W , Bramlett H . Therapeutic hypothermia and targeted temperature management for traumatic brain injury: experimental and clinical experience. Brain Circ. 2017;3(4):186‐198. 30276324 10.4103/bc.bc_28_17 PMC6057704

[66] Lutz Y , Loewe A , Meckel S , Dossel O , Cattaneo G . Combined local hypothermia and recanalization therapy for acute ischemic stroke: estimation of brain and systemic temperature using an energetic numerical model. J Therm Biol. 2019;84:316‐322. 31466769 10.1016/j.jtherbio.2019.06.011

[67] Kannojiya V , Das AK , Das PK . Numerical simulation of centrifugal and hemodynamically levitated LVAD for performance improvement. Artif Organs. 2020;44(2):E1‐E19. 31269235 10.1111/aor.13533

[68] Weber M , Steinle H , Golombek S , et al. Blood‐contacting biomaterials: in vitro evaluation of the hemocompatibility. Front Bioeng Biotechnol. 2018;6:99. 30062094 10.3389/fbioe.2018.00099 PMC6054932

